# Irrational choice and the value of information

**DOI:** 10.1038/srep13874

**Published:** 2015-09-09

**Authors:** Marco Vasconcelos, Tiago Monteiro, Alex Kacelnik

**Affiliations:** 1 School of Psychology, University of Minho, Campus de Gualtar, 4710-057 Braga, Portugal; 2 Champalimaud Neuroscience Programme, Champalimaud Centre for the Unknown, 1400-038 Lisbon, Portugal; 3 Department of Zoology, University of Oxford, Oxford OX1 3PS, UK

## Abstract

Irrational decision making in humans and other species challenges the use of optimality in behavioural biology. Here we show that such observations are in fact powerful tools to understand the adaptive significance of behavioural mechanisms. We presented starlings choices between probabilistic alternatives, receiving or not information about forthcoming, delayed outcomes after their choices. Subjects could not use this information to alter the outcomes. Paradoxically, outcome information induced loss-causing preference for the lower probability option. The effect depended on time under uncertainty: information given just after each choice caused strong preference for lower probability, but information just before the outcome did not. A foraging analysis shows that these preferences would maximize gains if post-choice information were usable, as when predators abandon a chase when sure of the prey escaping. Our study illustrates how experimentally induced irrational behaviour supports rather than weakens the evolutionary optimality approach to animal behaviour.

Reports of irrational behaviour, defined either as failure to maximize a well-defined benefit or as showing inconsistent preferences^1^, populate a growing catalogue of putative ‘cognitive biases’ for humans and other animals. Whilst these reports coexist with evidence for rational choice in other cases^2^, they serve as support for influential currents of behavioural and economic sciences^3^,^4^,^5^,^6^,^7^,^8^,^9^,^10^ and inspire objections to the relevance of the optimality modelling of behaviour that prevails in behavioural ecology. Irrationality is interpreted as reflecting cognitive biases or ad-hoc heuristics, but it can in fact help to understand the adaptiveness of decision processes in ecological circumstances, if psychological mechanisms and normative accounts of behaviour in natural problems are considered jointly.

Here we investigate an experimental protocol in which animals systematically display sub-optimal (irrational) behaviour: in a choice between two food sources, they prefer the option that yields lower probability of reward but richer information, even if such information cannot be used to alter forthcoming events. In our experiments, captive starlings (*Sturnus vulgaris*) chose between cues for either of two options. One option (*Info*) offered lower probability of reward, but informed about the forthcoming outcome immediately after being chosen, by displaying either stimulus X^+^ or X^−^, that respectively signalled sure forthcoming reward or sure absence of reward. The other option (*Noninfo*) offered higher probability of reward, but upon being chosen displayed either Y^0.5^_a_ or Y^0.5^_b_, both yielding equal probabilities of reward or its absence, so that the outcome was uncertain until it happened. In both options outcomes (reward or its absence) were realised 10 seconds after each choice but the duration of uncertainty was longer in the *Noninfo* option (Fig. 1a). This procedure is a variation of one developed by Zentall and collaborators^11^,^12^,^13^,^14^, working with pigeons (*Columba livia*), and we refer to it as the Z-protocol. Zentall and collaborators found that pigeons prefer *Info* when it yields a 20% chance of reward over *Noninfo* yielding a 50% probability. An absolute preference for *Info* implies foregoing 60% of the maximum achievable benefit, and a loss of 15% respect to random choice. This is a serious challenge for normative analyses. In a related protocol known as ‘observing response’^15^,^16^ subjects (including humans) also show willingness to pay a response cost to acquire information that cannot be used, but this phenomenon is less extreme because subjects do not actively choose a lower reward probability. Analogous findings have also been reported in the fields of economics and neuroscience^17^,^18^,^19^. Yet, similarly to the typical Z-protocol results, there is evidence that under some circumstances monkeys will reliably sacrifice amount of reward to obtain advanced information about the outcome of their choices^20^.

We examine the Z-protocol using the formalism of classical foraging theory^21^, that predicts preferences based on minimization of lost opportunity, or, equivalently, maximization of the ratio of expected gains to expected time. We then relate this analysis to plausible psychological processes and test novel predictions using experimental variations of the protocol. Our main assumption is that under natural circumstances any chase will be aborted if the predator is certain that the prey will escape whereas in the Z-protocol, information of certain no-reward cannot be used and thus the animal must pay the opportunity cost. Thus, the animal behaves in the laboratory with preference criteria that are adaptive in the field but misfire in some artificial circumstances.

## Optimal Foraging and the Z-protocol

In classical foraging theory the time paid pursuing behavioural alternatives is paramount, because rate maximizing models contrast expected gains from pursuing each alternative against the opportunity of using that time foraging elsewhere. In a foraging scenario parallel to the Z-protocol, after searching on average for a time *s*, a predator has the opportunity of pursuing either of two prey types of equal energy content (1 unit). Each type *i* has capture probability *p*_*i*_, and involves times *t* of pursuing and *h* of handling a prey (for simplicity we assume *t* and *h* to be equal across options and *h* to be zero when the prey escapes). To stress the parallel with the Z-protocol, we assume that all chases last the same (i.e. have the same opportunity cost) regardless of outcome. The returns (R_i_, in energy/time) that a predator gets if it chooses exclusively prey *i* is given by


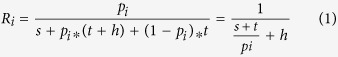


Across the full range of reward probabilities (0 < *p*_*i*_ ≤ 1)*, R*_*i*_ is a monotonic, increasing function of *p*_*i*_, with a maximum value of (*s* + *t + h*)^−*1*^. In the Z-protocol, *p*_*info*_* < p*_*noninfo*_, hence equation (1) predicts a preference for *Noninfo*, the opposite of what has been observed in pigeons. The (mechanistic) cause for the pigeons’ preference must be sought in the properties of the information processing mechanisms used by animals. In this case, since the contingencies are learned, it is relevant to relate the present problem to learning theory.

## The ITI and Learned Relative Valuation

In learning theory, arbitrary stimuli acquire power to modify behaviour (i.e. become conditioned stimuli, CSs) because they are contingent with biologically meaningful events such as food rewards (unconditional stimuli, USs). In widespread accounts of associative learning derived from the classic Rescorla-Wagner model^22^, information acquisition is structured in trials, without reference to temporal components such as *s* and *h* which play a major role in the foraging view. However, more recently some authors^23^,^24^,^25^ have taken an informational approach that gives a major role to temporal components, thus facilitating the integration between learning and foraging theories.

In informational accounts, learning about a reward-correlated stimulus depends on reward expectation in its presence relative to reward expectation in the context as a whole. The greater this ratio, the easier is learning, and the greater the stimulus’ asymptotic attractiveness. Similarly, in models of optimal foraging in patchy environments such as the Marginal Value Theorem^26^, travel time between patches influences hunting success in the environment as a whole, and consequently (through the effect of lost opportunity) the optimal exploitation policy for each patch. Consistently with both ideas, we express the attractiveness of an option as the ratio of reward expectation in its presence to reward expectation in the overall environment.

We define reward expectation in the presence of a stimulus *S*_*i*_ as 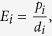 where *p*_*i*_ and *d*_*i*_, are the probability and delay to reward in the stimulus’ presence^27^. When multiple stimuli share a background, the attractiveness *A*_*i*_ of each one will be proportional to its expectancy relative to reward expectancy in the whole environment, as follows


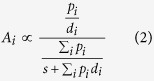


Notice that the denominator is common to all stimuli in the environment, and includes the expected searching time between encounters. A further issue is how to relate attractiveness (subjective value) to behaviour. This is an old but unresolved matter in decision studies. One view is that given different subjective value between options, subjects would follow the maximizing strategy of allocating all behaviour to the richer alternative. This behaviour however has costs if the environment is not stable, because exclusive allocation deprives the subject of information about alternatives that are never chosen. Empirically, partial preferences are frequently observed, and in particular it is frequently claimed that in many protocols preference between stimuli is proportional to (or at least approximated by) the ratio of experienced reward. If, as assumed under matching^28^, preference is determined by the ratio of attractiveness as defined in equation (2), then the denominator falls out of preference computations and so does the influence of *s*, which in the laboratory is equivalent to the inter-trial interval (ITI). In that case preference between two stimuli *S*_*1*_ and *S*_*2*_ is given by


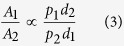


Thus, according to these assumptions we can expect foragers to be unaffected by *s* in equation (1) when facing choices between multiple sources of food, even if they do consider *s* when attributing value to each isolated stimulus. The analysis so far only takes into account reward probability and delay, but in the Z-protocol the options also differ in their informational properties. We now turn to the possible role of information.

If a foraging animal begins to chase a prey that immediately vanishes out of sight or becomes certain to escape, as it happens with probability 1-*p*_*info*_ in the *Info* option, an optimal forager would abandon the chase, avoiding the effort and loss of background foraging opportunity. A similar argument has been put forth in the aforementioned ‘observing response’ literature^15^,^16^, according to which engagement in a task can be weakened or lost when a stimulus predicts the absence of reward^29^. This means that in the denominator of the rate computation described by equation (1), (1-*p*_*info*_)*t = 0, so that in the *Info* option the experienced rate of reward for a consumer able to abandon purposeless chases would be


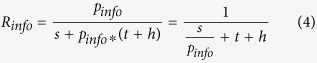


If preference *P*_*i,j*_ for option *i* respect to option *j* is determined by the ratio of the two expected profitabilities, (i.e. to connect the psychology of choice with the ecological perspective we substitute profitability for attractiveness) we get


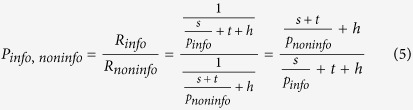


which, for an animal that is insensitive to the searching time *s*, reduces to


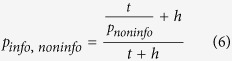


According to equation (6), *P*_info, *noninfo*_ > 1, for all 0 < *P*_*noninfo*_ < 1, namely *Info* is preferred against *Noninfo* regardless of reward probability in the informational option.

The result is interesting because: (i) it is counterintuitive; (ii) it derives from an integration between optimal foraging theory and the psychology of learning; (iii) it accounts for existing information about choice in pigeons; and (iv) leads to novel predictions that can be tested with further experiments.

Applying this rationale to the Z-protocol and denoting ≻ for “preferred”, the following relations are to be expected:

### Prediction 1: Effect of changing reward probabilities

Our preceding rationale leads to expect that *Info* ≻ *Noninfo*, regardless of reward probabilities.

### Prediction 2: Knowledge of terminal links

If, as assumed, subjects have learned the outcomes that follow the signals shown after their choice, and prefer higher probability of reward when allowed, the following preferences are expected:

X^+^  Y^.5^_a_, Y^.5^_b_

Y^.5^_a_, Y^.5^_b_  X^−^

### Prediction 3: Duration of uncertainty

In the Z-protocol the reward-collecting action in the *Info* option has a deterministic outcome and that in the *Noninfo* option has a probabilistic outcome. In addition, outcome uncertainty lasts longer in the latter. Since both humans and non-humans prefer early resolution of uncertainty^15^,^16^,^19^,^30^,^31^,^32^,^33^,^34^ it is useful to examine whether the duration of uncertainty or the predictability of the collecting action drives the results. If the protocol is modified so that the duration of uncertainty is equalised across options but the predictability of the collecting action is unaltered, the latter account predicts no change in preference, but the uncertainty duration one does. If duration of uncertainty is paramount, delaying the time at which information appears in the *Info* option would eliminate its advantage, and preference would follow reward probabilities.

### Prediction 4: Salience of the signal for sure reward

One mechanistic hypotheses aiming at explaining the observed paradox is based on psychological contrast. The idea is that positive surprises have greater hedonic value when they occur against a leaner expectation^35^,^36^. In the *Info* option, X^+^ causes elation because it is rare and increases the conditional reward probability from .2 to 1, while X^−^ does not cause much frustration both because it is frequent and then expected and because the change in conditional reward probability is smaller (from .2 to 0). We reasoned that if we maintain all probabilities but omit the signal for sure reward (i.e. do not have a physical stimulus for X^+^) the contrast effect would decrease, while interpretations based purely on expectation would be unaltered, because subjects would be able to infer that a reward is due from the absence of X^−^, even if no salient signal is presented after “lucky” choices. According to this informal reflection, if the Z-protocol is modified to omit X^+^, the contrast explanation would no longer cause the paradoxical preference for *Info*, while the reasoning underlying equation (6) is that this change should have no effect, leading again to *Info* ≻ *Noninfo*.

### Prediction 5: Sequential versus Simultaneous encounters

We have so far discussed simultaneous choice, but the logic of classical foraging as embodied in equation (1) and its modifications is more relevant when foragers encounter opportunities sequentially. In sequential encounters, rather than choosing between simultaneous signals for prey with different probability of escaping, predators opt between pursuing a potential prey or continue searching. In such scenarios, latency to start pursuing reward opportunities is a decreasing function of expected profitability relative to background opportunities; the decision to pursue richer options is taken faster than poorer ones^37^. It has been argued that latencies in sequential encounters are more mechanistically and ecologically meaningful than preferences in simultaneous choices, because the former predicts the latter, but not the other way round. The Sequential Choice Model^38^ (SCM) develops these ideas, which are well supported in the present study species. This rationale should apply to the Z-protocol. We test it below asking whether latencies in forced trials predict preferences in simultaneous trials even when the subjects favour low reward probability options.

These five predictions have different status. Predictions 1 and 3 follow from and test our foraging analysis, prediction 2 tests assumptions of that analysis, namely that paradoxical preference for low probability is not driven by the animals imperfect knowledge of the outcome of each signal, prediction 4 establishes a link to the psychology of contrast and prediction 5 links the present protocol to a related but different foraging analysis that nevertheless should apply here. We start by testing experimentally whether the paradoxical preference reported in pigeons is also present in our study species, the starling, and then modify the procedure to test these predictions and discuss their significance.

## Results

### Experiment 1

**Figure 1:**
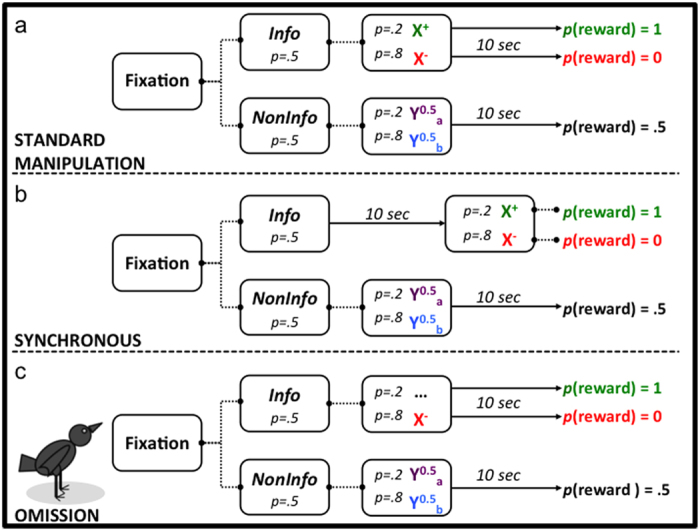
Design of Experiments 1 and 2. Dotted lines indicate no delay between consecutive events and solid lines indicate a 10 s delay. *p* denotes probability.

The first experiment (Fig. 1a) aimed to test Predictions 1, 2 and 5. In particular, we wanted (a) to examine some of the procedural ingredients responsible for the reported maladaptive choice pattern, paying particular attention to the knowledge starlings had about the signalling properties of the four terminal signals and the resistance of their preference to further reductions in the probability of reward in the *Info* option, and (b) to test whether maladaptive decisions can be anticipated from sequential encounters. To test prediction 5, we averaged latencies from the 64 single-option trials preceding each choice and predicted that the option with the shorter average would be chosen.

#### Test for Prediction 1

**Figure 2:**
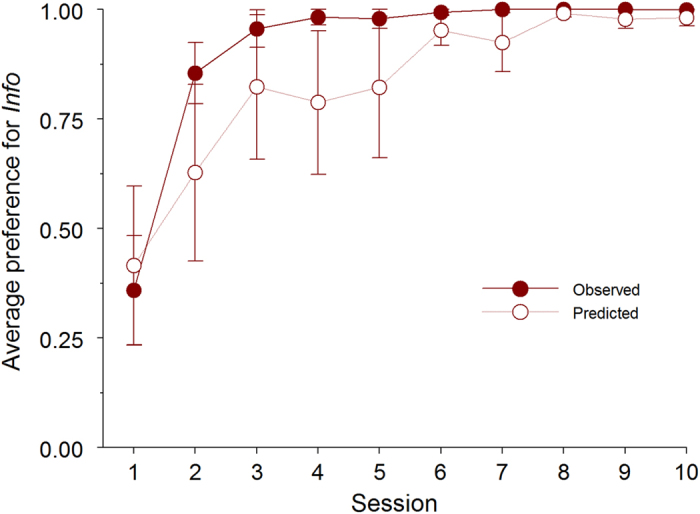
Average preference for the *Info* option in Experiment 1. Mean proportion of observed choices for the *Info* option in simultaneous presentations (±s.e.m.) and preferences predicted from latencies in no-choice trials according to the Sequential Choice Model (±s.e.m) across sessions in Experiment 1 (n = 6).

Figure 2 (full symbols) shows that starlings quickly developed a strong preference for *Info*. A repeated measures analysis-of-variance (ANOVA) confirmed a significant increase in preference for *Info*, as revealed by a significant effect of session (F_9,45_ = 24.335, *P* < .001). Pooled over the last 3 sessions, preference for *Info* reached 99.9% (s.e.m.: 0.0004; range: 99.8 – 100%). This was significantly above 50% (one-sample t_5_ = 93.444, *P* < .001), but not significantly below 100% (one-sample t_5_ = −1.000, *P* = .363).

**Figure 3:**
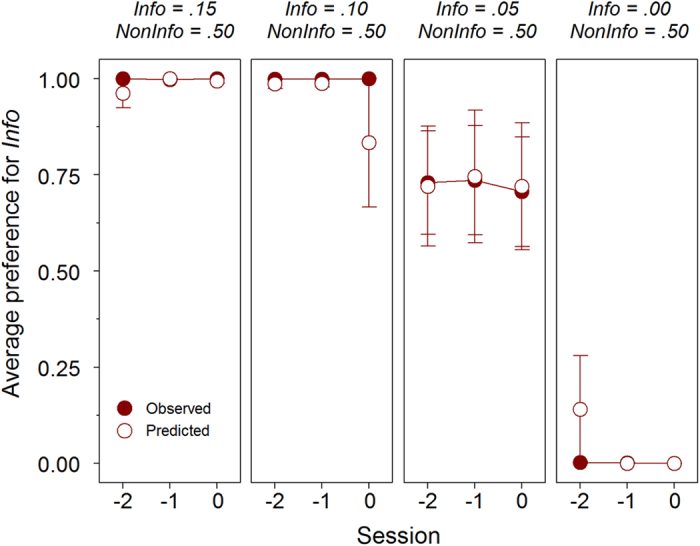
Average preference for the *Info* option with different reward probabilities in Experiment 1. Mean proportion of observed and predicted preferences according to the Sequential Choice Model (±s.e.m.) for the *Info* option during the last three sessions at each reward probability (n = 6).

Prediction 1 states that preference should be insensitive to *p*_*info*_. To test this we progressively decreased this parameter. Figure 3 displays preference for *Info* during the last three sessions of each value of *p*_*info*_ (full symbols). Preference for *Info* remained unaltered for *p*_*info*_ = 0.15 and *p*_*info*_ = 0.10. In the latter case this involved a loss of 80% of potential rewards. At *p*_*info*_ = .05, birds exhibited more variability and a mean preference for *Info* of about 75%, so that on average they lost around 67.5% of available rewards. In the control condition with *p*_*info*_ = 0.00, the birds reversed their preference, as might be expected. Averaging across sessions, preferences for *Info* were significantly above chance when *p*_*info*_ = 0.15 and 0.10 (t_5_ = 71.882, *P* < .001 and t_5_ = 74.738, *P* < .001, respectively), did not differ significantly from chance when *p*_*info*_ = .05 (t_5_ = 1.825, *P* = .128) and was significantly below chance when *p*_*info*_ = 0.00 (t_5_ = −55.974, *P* < .001). Thus, birds were almost insensitive to *p*_*info*_, showing behavioural changes only under absolutely extreme conditions.

#### Test for Prediction 2

**Figure 4:**
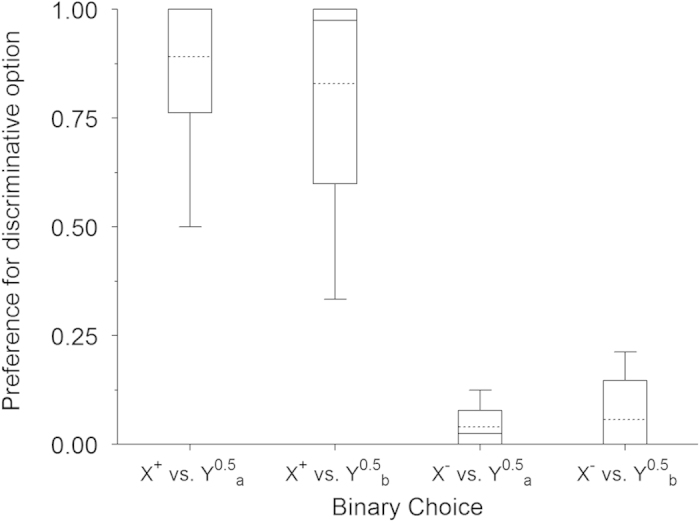
Average preference in choices between terminal stimuli in Experiment 1. The box plot includes medians (full horizontal lines), means (broken horizontal lines) quartiles (ends of the boxes) and extreme values (whiskers); n = 6.

The foraging analysis assumes that the observed irrational behaviour does not result from incomplete or distorted information about reward probabilities but from valuing information according to its potential to influence behaviour in the wild. It is however a possibility that what causes the paradoxical preferences is faulty learning or biased weighting of reward probabilities, similarly to assumptions embodied in Prospect Theory for human choice^39^. If they do learn the probabilities, preferences between terminal stimuli should appropriately reflect forthcoming outcomes. Figure 4 shows that when starlings’ were asked to choose between terminal links (in simultaneous presentations of the stimuli normally appearing after the choice) average preferences followed a rational ordering according to reward probability. X^+^ preference over Y^0.5^_a_ and Y^0.5^_b_ were 89.2% ± 0.082 and 82.9% ± 0.111 s.e.m., respectively. In contrast, preference for X^−^ against Y^0.5^_a_ and Y^0.5^_b_ were 3.96% ± 0.021 and 5.63% ± 0.037 s.e.m., respectively. All these preferences deviated significantly from chance (t_5_ = 4.377, *P* = .007; t_5_ = 3.016, *P* = .030; t_5_ = −9.889, *P* < .001 and t_5_ = −7.179, *P* = .001, respectively), thus confirming that preference for the *Info* option was not due to lack of knowledge of the relevant probabilities.

#### Test for Prediction 5

According to SCM, latencies in no-choice trials should correlate with preference in choice trials. Figures 2 and 3 (empty symbols) show that choice preferences were closely predicted from such latencies, both in the original condition (Fig. 2) and in subsequent tests with lowered values of *p*_*info*_ (Fig. 3). In Figure 2, predictions and preferences can also be seen to covary through acquisition, albeit showing a degree of temporal mismatch.

#### Discussion

The starlings quickly developed a strong preference for the leaner, informative option, and showed resistance to change despite substantial reductions in the probability of reward. Their preferences between signals that differed in information (initial links) meant foregoing up to 60% of available rewards, but their preferences between the terminal links were rationally ordered according to their objective properties. Preferences are thus not explained by subjective distortions or weighting of the reward probabilities corresponding to the terminal stimuli, but are consistent with the valuation influence of information, regardless of its usability. Throughout all these comparisons, preference in choice trials were well predicted by latency to accept each option in no-choice trials, providing further evidence in support of SCM, whose rationale depends on assuming that foraging decision mechanisms are adapted to sequential rather than simultaneous choices.

### Experiment 2

This experiment tested predictions 3, 4 and 5 using three groups of subjects.

Prediction 3 concerns whether the duration of uncertainty or the predictability of the collecting action drives the observed preferences. If the duration of uncertainty is equated between options, but the response preceding the outcome maintains the original contingencies, then under the first hypothesis the paradoxical preference should vanish, but under the second it should persist. In the Z-protocol (Fig. 1a), reward uncertainty vanishes immediately after choosing *Info* due to the onset of either a signal for safe reward or for sure no-reward, while in *Noninfo* the signals appearing after the choice are uncorrelated with reward and then uncertainty lasts 10 s longer. Thus the options differ in signals’ correlation and in the duration of uncertainty. Our foraging explanation for the bias towards *Info* relies on the duration argument, because in the transformation from equation (1) to equation (6), the time waiting for no-reward under certainty is edited out from the relative rate computation (as is, for different reasons, the ITI). We reasoned that if *Info* were modified so that correlated signals were still present but timed such that uncertainty lasted the same as in *Noninfo*, then all waiting times after choice would influence preference, which should now reflect reward rates. This was implemented in the Synchronous Group (Fig. 1b). If uncertainty duration is crucial, birds in this group should prefer *Noninfo*, because the waiting times would enter in the computation, even if the reward-collecting response has a predictable outcome.

Prediction 4 pitches the rationale leading to equation (6) against a psychological contrast mechanism based on the hedonic impact of the signal for sure reward or certain no-reward. To test these ideas we designed the Omission Group (Fig. 1c), which preserves the probability structure of the Z-protocol (Fig. 1a) but without a signal for sure reward. Under the logic leading to equation (6) preference for *Info* should survive, but under the contrast-dependent, signal salience idea the subjects should now prefer *Noninfo*.

The Control Group replicated Experiment 1. We examined whether latencies in forced trials predicted preference in simultaneous choices (Prediction 5) in all groups.

#### Tests for Predictions 3 and 4

**Figure 5:**
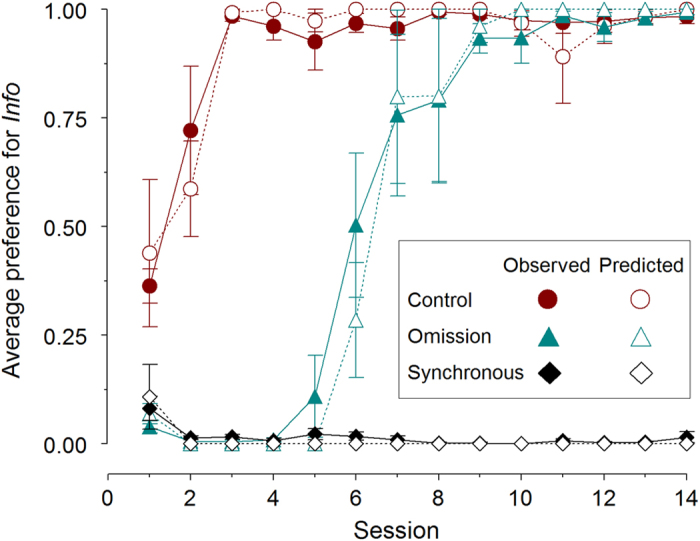
Average preferences for the *Info* option in Experiment 2. Mean proportion of observed and predicted preferences according to the Sequential Choice Model (±s.e.m.) for the *Info* option for the Control (n = 6), Omission (n = 5), and Synchronous (n = 6) groups in Experiment 2.

Figure 5 (full symbols) shows the acquisition of preference for the three groups over the 14 sessions. The Control Group (filled circles) reproduced the results observed in experiment 1. The Omission Group (filled triangles) initially showed a strong preference for *Noninfo*, but after a few sessions preference switched, reaching the same asymptotic preference for *Info* shown by the Control Group and previous results with the Z-protocol. It would appear that the typical, paradoxical result develops once the subjects learn the contingencies. It is notable that as the birds learned, their behavioural allocation became progressively more irrational for the local circumstances. The Synchronous Group (filled diamonds), which experienced equated durations of uncertainty, but where outcome probability after responding to the terminal links were just as in the standard Z-protocol, showed an almost exclusive and stable preference for *Noninfo*. In this group the asymptotic behaviour did maximize reward rate.

A mixed-design ANOVA with session and group as fixed factors and subjects as random factors confirmed these descriptions, yielding a significant main effect of group (F_2,13_ = 69.877, *P* < .001), session (F_13,169_ = 36.817, *p* < .001) and a significant interaction (F_26,169_ = 31.043, *P* < .001).

Pooled over the last three sessions, the average preferences for *Info* were virtually absolute for the Control and the Omission groups (97.9 ± 0.02 and 97.7% ± 0.016 s.e.m., respectively), but the exact reverse for the Synchronous group (0.007% ± 0.005). A one-way ANOVA on these asymptotic preferences revealed a significant effect of group (F_2,16_ = 291.893, *P* < .001), with post-hoc Scheffe’s tests confirming that preference for *Info* in the Synchronous group was significantly below that observed for the two other groups (largest *P* < .001). Asymptotic preferences for *Info* were so extreme that they were statistically undistinguishable from 100% for the Control and Omission groups (t_5_ = −1.225, *P* = .275; t_4_ = −1.790, *P* = .148, respectively) and from 0% for the Synchronous group (t_5_ = 1.497, *P* = .195).

#### Test for Prediction 5

Also shown in Figure 5 (empty symbols) are the average SCM predictions (preference in choice trials predicted from no-choice latencies). As in previous experiments, the SCM predictions match observed preferences in the three groups. Further, in the Omission group, where there is a strong temporal evolution including a reversal of preference as a function of experience, the SCM predictions track these changes closely.

#### Discussion

The observed patterns of choice replicated the findings of Experiment 1 in the Control Group, showed a delayed emergence of preference for *Info* in the Omission Group, and a strong preference for *Noninfo* in the Synchronous Group. This last result holds the key to understanding the sub-optimal choice observed in the standard Z-protocol. The key difference between cases with nearly optimal and grossly suboptimal preferences seems to be the timing of the removal of uncertainty. When uncertainty disappears at the same time in both options, as in the Synchronous group, subjects show an almost absolute preference for the high probability alternative. When instead the change in subjects’ information status in one of the options occurs immediately after the choice, the waiting time for certain no-food (i.e. during X^−^) seems to play no role in that option’s valuation.

Finally, in all conditions the latencies to accept each option in no-choice trials accurately and quantitatively predicted preferences in simultaneous choice trials, tracking changing preferences as learning proceeded. This provides strong evidence in support of the SCM.

## General Discussion

Experimentally proven deviations from rational or optimal choice are often included in critiques of the normative approaches to the study of behaviour prevalent in evolutionarily-inspired behavioural ecology^21^ or in axiomatic microeconomics^40^. The logic of critics is not limited to observed failure of normative predictions, but emphasizes that optimization involves computations that are too hard to make for organisms behaving in real time. For instance, supporting Herbert Simon’s program, Gigerenzer and Selten wrote “*The theory of bounded rationality, as we understand it, dispenses with optimization, and, for the most part, with calculations of probabilities and utilities as well*”^41^ ^(p 3)^. Similarly, in the same volume and for similar reasons, Klein says “*optimization should not be used as a gold standard for decision making*”^42^ ^(103)^. These arguments are valid if addressed to the processes controlling the agent’s behaviour, but are not relevant regarding the optimality models used by biologists to predict and/or explain what organisms do in nature as a consequence of mechanisms designed by natural selection. Here we defend optimality in the latter context, sustaining that deviations from the predictions of such models provide raw material to develop and improve them, and that calculations involving probabilities and utilities are a fundamental aid to the research program.

We first formally examined an experimental protocol (the Z-protocol) in which originally pigeons^11^,^12^,^13^, and now starlings (this study) incur major foraging losses by preferring an option that delivers certainty of reward or no-reward over a non-informative alternative where uncertainty remains until the outcome is realised. This analysis shows that “irrational” preference is to be expected if animals do not include two temporal components of the foraging cycle, the searching times (or ITIs) common to all options in the environment and the time waiting under certainty of no-reward. We also show that neglect of both time elements is to be expected when learning mechanisms are considered. Our approach is consistent with Simon’s “two-blades” well-known metaphor arguing that decision mechanisms are best understood when taking into account their interaction with the structure of natural choices. We are also consistent to some extent with the ecological rationality stand taken by Gigerenzer and his colleagues, but we do defend the use of calculations of probabilities and utilities because we take into account that psychological mechanisms are designed by natural selection across generations, and do not face the problem of computing optima in real time. Psychological mechanisms are in fact equivalent to the heuristics proposed by the bounded rationality school but are not dedicated to solving specific problems.

Regarding the learning process, we showed that if learning depends on expected reward in the presence of a stimulus relative to expected reward in the overall context, as assumed in (informational) learning theory, then the relative strength of signal-outcome association will be unaffected by temporal components shared by all stimuli in a given context. Examples of shared time components are the ITI in the laboratory and its equivalent of inter-patch intervals in the wild. We further reasoned that there are substantial and instructive differences between the Z-protocol and foraging in the wild. In the wild, information about a prey’s probability of escape acquired at any time during a chase is valuable to the predator, because the chase will be aborted if the prey is sure to escape, with effort (time) being reallocated to foraging elsewhere. In the Z-protocol, information about sure no-reward, equivalent to certainty that the prey will escape, cannot be used to redirect foraging effort. Thus, in the wild, an informative prey type would only cause opportunity cost when it is due to be captured, but in the Z-protocol a subject informed that reward is not forthcoming pays the same opportunity cost as in rewarded trials. Indeed, waiting time for sure no-reward is what makes the observed preference paradoxical. The structure of natural choices thus may not lead to the evolution of the ability to include time waiting for sure no-reward in the rate computations, simply because such time cost is never paid. This rationale served to make four novel predictions.

A further difference between the structure of natural foraging and the laboratory situation is that, as argued elsewhere^38^, most foraging in the wild is likely to entail sequential encounters with prey that can be pursued or ignored (closer to a go-no go protocol), while in the Z-protocol choice is between simultaneously encountered alternatives. Previous experimental work has shown that in sequential encounters the latency to respond is inversely related to how profitable each prey type is relative to the context, and that this latency is more informative than preference in simultaneous choices; the latter can be inferred from the former, but not otherwise^37^,^38^. As a fifth prediction we used relative latency to respond in no-choice trials as a predictor of preference shown in simultaneous choices, and tested this quantitatively in all our experiments.

All five predictions were supported by the experimental results. Most importantly, the paradoxical preference for the low-probability, informative option disappeared when the information in this option was programmed so that time under uncertainty was equalised, indicating that duration of uncertainty was responsible for the effect.

Our conclusion is that the irrationality observed in the Z-protocol results from testing the animals in a situation where information is useless, while the birds’ psychological processes are adapted to a world in which information alters the subsequent behaviour. This enhances the interest and scientific value of the protocol, when framed within an optimality analysis of decision making. As evolutionary biologists, we aim at bridging the gap between functional and mechanistic accounts of behaviour, and for this reason we disagree with Gigerenzer and Selten’s^41^ view that optimality competes with studying the “adaptive tool box” of real organisms, and that probabilities and utilities can be dispensed with. The biological optimization agent is natural selection, not the real-time behaving organism, and optimality is the tool used by biologists to unravel these links.

## Methods

### Subjects

Subjects were twenty six wild-caught adult European starlings (*Sturnus vulgaris*) with previous experimental histories. Eight participated in Experiment 1 and 18 in Experiment 2 (6 per group). During the experiments, starlings lived in pairs in indoor cages where they were visually, but not acoustically isolated. Each room contained two cages that served both as home and experimental cages. Indoor temperatures ranged from 15 to 18°C and lights followed a 12:12 light:dark schedule with light on from 0700 to 1900, and gradual transitions at dawn and dusk.

After the daily experimental sessions, starlings had four hours (1300–1700) of free access to turkey crumbs and Orlux^©^ Remiline universal granules, as well as 10 mealworms (*Tenebrio sp.*), and social interaction with the cage partner. This regime maintains starlings at approximately 90% of their free-feeding weight^43^ and provides social enrichment. When not participating in an experiment, they were housed together in two outdoor aviaries, with ad libitum food (a mixture of turkey crumbs, Orlux pellets and mealworms). Drinking and bathing water was always available and replaced daily. All subjects were released into the wild after participating in three experiments, and following at least two weeks of re-acclimatization to natural light in the outdoor aviary.

One bird was removed from Experiment 1 due to an injury and another revealed a systematic side bias and was thus excluded from all analyses. One bird from the Omission group was removed from Experiment 2 due to illness.

### Apparatus

**Figure 6:**
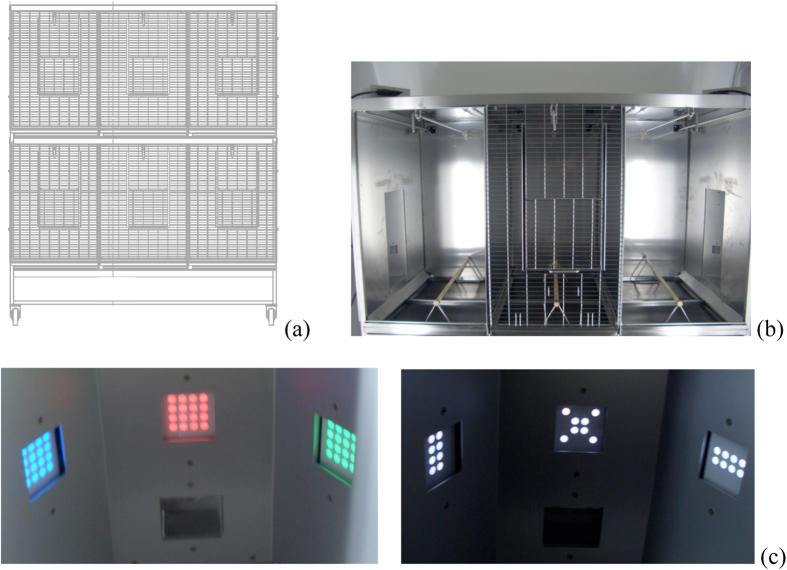
Experimental cages and panels. (**a**) Technical drawing of the experimental cages, capable of housing two birds in each vertically mounted unit. (**b**) Photograph showing top unit of an experimental cage. Units had two individual working areas on the sides, and a shared area in the centre. (**c**) Sample photographs of the pecking keys. Each key is composed of a 16 LED matrix capable of displaying different combinations of colours (left) and/or symbols (right).

Cages serving as home and experimental chamber for pairs of individuals (Fig. 6) were composed of two units, vertically mounted [135 cm × 78.4 cm × 80 cm (l × w × h) each]. Each unit included two experimental areas that were isolated during experimental periods so that subjects could be tested individually. These areas were separated by a common middle section, so that outside experimental time the two individuals in each unit shared a larger space where they could fly freely. Each individual experimental area [45 cm × 78.4 cm × 80 cm (l × w × h)] had a 40 cm tall panel, attached 10 cm above the floor, with three sections, all 11.5 cm wide: a middle sub panel, facing the cage, and two side ones at 120 degree angles from the central subpanel. Middle subpanels had one response key in the centre (11 cm from the bottom), and a food hopper (2.5 cm from the bottom) connected to a pellet dispenser (Campden Instruments®) containing 20 mg BioServ® precision pellets. Each side subpanel had one response key in its centre (11 cm from the bottom). Behind each response key there was a 16 LED light matrix that could display 16 different symbols in seven possible colours. A computer in an adjacent room controlled all experimental events and recorded data.

### Experimental Protocol

#### Preliminary training

Starlings from both experiments received preliminary training sessions in which they had to peck for food on each side key to one of five hues (white, red, green, orange and lilac; all 4 × 4 LEDs), counterbalanced across trials, as well as to a white X symbol (8 LEDs) on the centre key.

#### Experiment 1

Subjects were exposed daily to two types of trials: single-option (n = 280) and choice (n = 140) trials. Single-option trials involved the presentation of either the *Info* or the *Noninfo* option in one of the side keys (140 trials each). For each starling, *Info* was always presented in one of the side keys and *Noninfo* in the other, with side allocation counterbalanced across birds. These trials always began with flashing (700 ms ON, 300 ms OFF) of the centre attention key (always with a white X symbol). A peck to the centre key turned it off, and caused one side key to be illuminated in white, with side indicating whether it was an *Info* or *Noninfo* trial. In *Info* trials a single peck to the side key turned it off and caused that key to switch to one of two terminal hues (for instance, red or green), depending on whether that trial was to end with reward (X^+^) or no-reward (X^−^) which occurred with probabilities *p*_*info*_ = 0.2 and (1-*p*_*info*_) = 0.8, respectively. The terminal hue was automatically turned off 10 sec after onset and was followed by delivery of two precision pellets when X^+^ was presented and nothing (i.e. entering a new ITI) when X^−^ was presented. The terminal hues on *Info* trials were discriminative, because they signalled forthcoming food or no food without ambiguity. In *Noninfo* trials, a single peck to the side key caused it to switch to either of two different terminal hues (for instance, orange or lilac), with probabilities 0.2 (Y^0.5^_a_) and 0.8 (Y^0.5^_b_). The terminal hue was turned off 10 sec after onset, but both hues were followed by food with probability *p*_*noninfo*_ = 0.5. Thus, these terminal hues were non-discriminative. The terminal hues associated with both options as well as their assignment to the low and high frequency of occurrence were counterbalanced over subjects.

In choice trials, after pecking the attention key, both side keys were illuminated in white. A single peck to either key turned the alternative key off, and switched the selected key to one of the terminal hues as for single-option trials. Single-option and choice trials were randomly interspersed. Trials were separated by a 25 sec ITI. Daily sessions started at 0730 and ended at 1300 or when 420 trials were completed, whichever came first.

After 10 sessions as described, for 4 further sessions we interspersed (in addition) 20 terminal link choice trials. In these trials pecking the centre key was immediately followed by simultaneous presentation of the following stimulus pairs: X^−^ vs.Y^0.5^_a_, X^−^ vs. Y^0.5^_b_, X^+^ vs. Y^0.5^_a_, and X^+^ vs. Y^0.5^_b_, five of each. In terminal link trials subjects expressed preferences between terminal links rather than between the *Info* and *Noninfo* options. A peck turned the unselected key off and delivered the normal contingency to the selected key.

In the final phase of the experiment, the profitability of *Info* was reduced by lowering *p*_*info*_, namely the probability of X^+^, in steps from the original 0.20 to 0.15, 0.10, 0.05 and finally 0.00. Subjects received a minimum of 5 sessions per probability level and continued on a given condition until they showed stable preferences between *Info* and *Noninfo*. Stability was defined by 3 consecutive sessions showing no trend in the proportion of choices, and a standard deviation in this proportion of less than 0.10.

#### Experiment 2

Training was similar to Experiment 1, except that a peck to the terminal stimulus was required to initiate the 10-s delay in both options of the Control group and the *Noninfo* option of the Synchronous group. A peck the terminal link of the *Info* option of the Synchronous group terminated the stimulus. Apart from these differences, the Control group was trained as described Experiment 1 (cf. Fig. 1a). A schematic of the design for the remaining two groups (Synchronous and Omission) is presented in Figures 1b,c. These groups differed from the Control only in *Info* trials. For the Omission group, X^+^ was omitted, but on 20% of the *Info* trials a single peck to the white side key turned it off and food was delivered after 10 s. On the remaining 80% of *Info* trials, X^−^ was presented and the trial ended with no food after 10 s. For the Synchronous group, the 10-sec delay occurred between the choice of *Info* and information being given. Thus, when *Info* was illuminated, the first peck initiated a 10-s delay. Once the delay lapsed, the key was turned off and either option X^+^ or X^−^ was shown. All birds received 14 sessions.

### Data analysis

Prior to analysis, all proportion and latency data were successfully normalized using an arcsine square root and a natural log transformation, respectively^44^. A Type-1 error rate of 0.05 was adopted for all statistical comparisons. The SCM’s predictions were calculated using latencies from single-option trials preceding choice trials. To minimize the influence of temporal fluctuations in the subject’s state spanning over groups of trials, we used average latencies collected in the 64 single-option trials preceding each choice (which in some cases included trials from the preceding sessions) as a metric of value for each option^38^. The predicted choice was the option with the shorter average latency. This approach entails using the same latencies to predict more than one choice, but there is no reason to suspect that any effect of this should bias predictions one way or another.

### Ethical Note

All experiments were approved by the Department of Zoology Ethical Committee, University of Oxford, and were carried out in accordance with the current laws of the United Kingdom. Animals were obtained under English Nature license No. 20010082 and were cared in accordance with the University of Oxford’s “gold standard” animal care guidelines.

## Additional Information

**How to cite this article**: Vasconcelos, M. *et al.* Irrational choice and the value of information. *Sci. Rep.* **5**, 13874; doi: 10.1038/srep13874 (2015).
